# Exploring the Gender-Related Perceptions of Male Nursing Students in Clinical Placement in the Asian Context: A Qualitative Study

**DOI:** 10.3390/nursrep11040081

**Published:** 2021-11-01

**Authors:** Yuk-Chiu Yip, Ka-Huen Yip, Wai-King Tsui

**Affiliations:** School of Health Sciences, Caritas Institute of Higher Education, Hong Kong 999077, China; khyip@cihe.edu.hk (K.-H.Y.); ztsui@cihe.edu.hk (W.-K.T.)

**Keywords:** nursing education, clinical learning, male nursing students

## Abstract

The lack of gender diversity in the nursing profession has long been recognized as a cause for concern. Female nurses in many practice settings across nations continue to outnumber their male counterparts. Male nursing students may have perceived differences in the way they are treated in clinical practice; however, limited studies have been conducted to determine the unique perspectives of these students in terms of gender bias in their clinical learning. To address this knowledge gap, this study employed a qualitative descriptive approach to explore the experiences of male nursing students in clinical learning in an Asian context. Specifically, we examined the insights emerging from the thoughts and feelings of male nursing students from interactions with their clinical mentors, patients, and peers in their encounters during their clinical placement. From November 2019 to July 2020, data were collected through semi-structured interviews from 22 male participants aged 21–30 years (mean age: 22.7), enrolled in a prelicensure Bachelor of Nursing (honors) program in Hong Kong. Four themes emerged after a qualitative content analysis: (1) while the School of Nursing welcomes men, clinical settings are another story due to pragmatic considerations; (2) you are welcomed because you may be seen as a helper with greater physical strength; (3) male nursing students feel alienated in the obstetrics and gynecology practicum; (4) more male role models are desired in the clinical setting. Our findings indicate the need to promote gender awareness among faculty and clinical mentors, understand the factors hindering and facilitating the clinical practicum for male nursing students, improve the male nursing experience during the maternity practicum, and ensure access to more male role models to boost role socialization and learning.

## 1. Introduction

The World Health Organization [1] indicates that only 10% of nursing professionals worldwide are male, with some variation in rates across regions and countries: 10.6% of nurses in the UK are male, while the figures are 10.9%, 1%, and 12.7% in Australia, China, and Hong Kong, respectively [2,3]. Males have reported a range of reasons for choosing to train as nurses; similar to their female counterparts, many cite an altruistic desire to undertake work of a caring nature [4]. In many cases, however, it is not explicitly expressed as “caring;” instead, it is described as a meaningful way to achieve professional fulfillment [5].

Despite this similarity in terms of motivation for entering the profession, male nursing students have perceived differences in the way they are treated in clinical practice, which may be attributed to gender stereotypes [6,7]. Studies have shown that many male nurses are perceived as providers of “muscle” to lift or move patients, or are called in as security back-up in case of possible violence [6]. Others have felt particularly unwelcome when undertaking clinical training in maternity units, either due to a lack of support from clinical instructors or restrictions on what they are asked to do and learn [8]. Further instances of gender bias include male nurses being assigned to observe rather than undertake hands-on roles, being asked to leave a bedside even when the patient did not request it, and being excluded from labor wards and neonatal units [9]. Furthermore, although multiple studies have found that male nurses wish to be addressed and referred to simply as “nurses” in clinical settings, the adjective “male” is routinely affixed—partly because it is commonly assumed among patients that the males in the wards must be doctors [7]. Indeed, frequent accounts have been given of patients asking male nursing students, but not females, whether they are going to qualify as physicians after completing their nursing studies [7].

The current study aimed to explore the male students’ perspectives on gender bias from their clinical learning experiences toward pursuing a baccalaureate prelicensure nursing program in Hong Kong. Our research team adopted a qualitative descriptive approach because this method may generate insights emerging from the thoughts and feelings of male nursing students from interactions with their clinical mentors, patients, and peers in their encounters during their clinical placement. This study has important practical implications. As nursing staff shortages may intensify with an increase in the elderly population of different nations, the barriers as perceived by male nursing students warrant attention from not only the faculty within School of Nursing but also the management, staff, and mentors in diverse clinical areas. The findings from this study may yield information that allows nurse researchers and educators to determine whether gender biases (which may have been discussed in the literature) persist at the time this research. Further, the concerns of students could be addressed with a view to providing an inclusive workforce [10]. As a profession in which females in many practice settings (such as in Hong Kong) outnumber males, the retainment and recruitment of male nursing students should first start with a facilitative learning atmosphere. Through this study, we hope to enhance the awareness of practicing nurses of the needs and concerns of male nursing students. A clinical learning environment with no gender bias is vital for future nurses.

## 2. Methods

This study used a qualitative descriptive approach to examine the experiences of male nursing students in clinical placement in Hong Kong. Data were collected through semi-structured interviews with 22 male nursing students and analyzed through qualitative content analysis.

### 2.1. Study Setting and Population

This study was conducted at a higher educational institute in Hong Kong which provides a full-time five-year baccalaureate nursing program leading to an academic qualification recognizable by the Nursing Council of Hong Kong. Students who complete the program satisfactorily are eligible to be registered through the Council to be practicing as registered nurses (general) in Hong Kong. Study participants were recruited by the researchers (YC, KH, and WK) based on the following inclusion criteria: (a) ability to understand and speak Cantonese; (b) currently enrolled in the said full-time baccalaureate program; and (c) completed at least 25 days (200 h) of clinical practicum. The exclusion criteria were: (a) female nursing students; and (b) nursing students who completed less than 25 days of clinical practicum (this threshold [days] was set because in the program, the first 20 days of practicum are closely mentored by nursing teachers in the school; students spending more placement hours independently in clinical areas were desired by the research team because they may be able to share richer experiences).

### 2.2. Sampling

This study utilized convenience sampling, a non-probability sampling method where participants are conveniently selected to participate in this study. From November 2019 to July 2020, eligible male nursing students were approached in person by the research team. After recruiting interested male nursing students, the researcher (YC) arranged to interview them. All nursing students who met the inclusion criteria were invited to participate in this study to enhance the diversity and richness of the information gathered [11].

### 2.3. Data Collection

The researchers (YC and KH) explained the information sheet and interview process to the participants before asking them to sign a written consent form. Informed written consent was obtained from them to ensure their right to full disclosure. With the use of interview guides (see Table 1), one researcher (YC) facilitated each in-depth interview, while two others (KH and WK) took field notes, documenting significant observations from the participants’ physical expressions and gestures to enhance the examination of the participants’ verbal and nonverbal information. The interviews were also audio recorded. Our research team (YC, KH, and WK) determined that no new information emerged after 20 interviews were conducted (indicating that data saturation was reached [11]).

The interviews were held in places that were convenient for the respondents (usually a private study room in the library or a small classroom at the campus). Depending on the depth of the discussions, each interview duration was approximately 45–60 min (mean duration: 55 min).

### 2.4. Data Analysis

After collecting data, two researchers (YC and KH) transcribed the interviews verbatim to ensure the accuracy of the transcription. The first researcher (YC) translated the interviews verbatim from Cantonese to English, while the second (KH) checked the accuracy of the transcription. The researchers (YC, KH, and WK) then analyzed the translated interviews through qualitative content analysis [12]. Content analysis was primarily conducted through identifying meaning units, creating categories, and developing schemes of categorization [12]. Specifically, during the coding process, the transcripts were read and analyzed independently by each researcher (YC, KH and WK). The interviews were read several times to understand the full context. The text regarding the participants’ experiences in clinical practicum was extracted and compiled into one text by researchers (YC and KH), which constituted the unit of analysis. Subsequently, we compiled the themes and sub-themes that arose from the interviews [13]. The NVivo software (NVivo Version 11, QSR International, Victoria, Australia) was used to facilitate the content analysis.

### 2.5. Ethical Considerations

Ethical approval was obtained from the Research and Ethics Committee of a higher education institution prior to the commencement of this study (reference number HRE210112). The work in the research was carried out in accordance with the Declaration of Helsinki. All participants were guaranteed anonymity and confidentiality. Participation was entirely voluntary, and the participants had the right to withdraw from this study at any time without any negative consequences.

### 2.6. Rigor

Credibility. Peer debriefing, member checking, and interview techniques were adopted in this study. Regarding peer debriefing, the researchers (YC and KH) discussed the research process and interview guide designed based on existing literature [14,15,16] with two university professors experienced in conducting qualitative studies in the field of nursing education. Their feedback helped improve the inquiry process and the overall quality of findings. The researchers (YC) also used member checking in analyzing and interpreting data, to reduce bias and inconsistency in the results [13].

Dependability. Dependability was established using stepwise replication, a code–recode strategy, and triangulation [13]. Stepwise replication was used as the evaluation procedure [13]. The research team (YC, KH, and WK) analyzed the data separately then compared the results [17]. Coding was performed by YC after all verbatim transcriptions were verified by KH and WK. The same data were re-coded to detect any inconsistencies in the first coding [13]. Finally, triangulation was used to reduce bias. The integrity of the participants’ responses was also examined. Since multiple researchers (YC, KH, and WK) were involved in investigating the topic, different perspectives were brought to the research, which strengthened the findings [13].

Confirmability. Confirmability was achieved via audit trails, facilitating data validation, and field notes, which were used to note nonverbal behavior and conversations. The researchers (YC and KH) conducted a thorough audit trail to cross-check the inquiry process: field notes, documents, and records collected from the field [17]. Thereafter, field notes were kept by the research team (YC, KH, and WK) to reflect on, interpret, and plan the data collection and analysis. All events transpiring in the field during the interview were maintained [13].

Transferability. The researchers provided a detailed description of the inquiry. This facilitates the transferability of the inquiry.

## 3. Findings

A total of 22 male nursing students aged between 21 and 30 (mean age: 22.7), with an average of 889 completed hours of clinical practicum, participated in this study. Sixteen were in the fifth year of their studies while six were in the fourth year. All were unmarried, of Asian descent, and fluent in English. One had obtained a bachelor’s degree in business before entering nursing education. The participants from the current study did not have previous experience working in a health care setting. From the data analysis, four themes emerged from the nursing students’ experiences in clinical learning.

### 3.1. While the School of Nursing Welcomes Men, Clinical Settings Are Another Story Due to Pragmatic Considerations

In general, the participants felt welcomed at the School of Nursing across both classroom and workshop environments. They emphasized that even when the subject matter appeared oriented to areas wherein female nurses were more likely to work, such as pediatrics and obstetrics, teaching staff did not use gendered references or stereotyped assumptions in the classroom. Participant 3, for example, observed the following:

“They’re really good about not making gender an issue in school, even when we are learning about women’s health. But when we are in the wards, our mentors frequently don’t use inclusive language. To the extent that I’ve often heard mentors explain a nursing procedure telling the male students that it doesn’t matter whether they understand it or not, because we’re going to have to ask for help from female nurses in the future anyway.”

The participants felt that their instructors deliberately avoided gendered references and assumptions in both the classroom and clinical education. Participant 4 noted that teaching staff were “welcoming and inclusive,” while Participant 2 observed that they used gender-inclusive language, being careful not to refer to a nurse as “she” during teaching sessions, which he felt was a good practice as there are many male nurses in Hong Kong. Participant 7 had a more nuanced view, although he perceived it as pragmatism rather than prejudice.

“I am not saying that male nursing students are rejected in clinical settings, but it does seem that nurses take a pragmatic point of view. Female nurses tend to think that when they are working in a mixed ward (where there are both female and male patients), the wisest course is to get the male students to focus on caring for the male patients to protect them just in case ungrounded complaints are made by the female patients.”

Participant 14 also emphasized the pragmatic nature of occasional gender-biased practices.

“Female nurses in clinical areas think that the best strategy for gender-related issues is to tell male students to focus on looking after the male patients. I guess female nurses commonly think like this because every shift in every ward has mostly female nurses. It’s just not wise to put a male student in a potentially risky situation.”

Participants (2, 7, 14, 15, and 17) noted that nurses actively took measures to avoid potential difficulties due to gender differences. Although nurses did not say so explicitly, the pattern of tasks assigned to male students clearly suggested that the staff or mentors were operating based on pragmatic considerations—that protecting students means protecting themselves from any patient complaints. The participants emphasized their understanding of this pragmatic method of doing things, stating that when it is their turn to mentor nursing students in the wards, they will also operate according to the maxim of “don’t go looking for trouble” (participants 2, 7, 14, 15, 17).

### 3.2. You Are Welcomed Because You May Be Seen as a Helper with Greater Physical Strength

While all participants did not object to performing heavily physical duties, such as lifting patients, on most occasions, they did not appreciate being taken out of the learning environment just because “muscles” were needed, as they could miss important learning opportunities. Participant 10 stated:

“I am totally fine about being perceived as physically stronger, being called on to bathe patients and transfer them between stretchers and beds. The problem is that once male students are widely seen as principal providers of muscle in nursing, then certain mentors, particularly the female ones, take it for granted that this is what we’re supposed to do. During important learning opportunities and vital procedures like assisting with a bone marrow biopsy or caring for central lines, they will still tell you to leave the observation and go off to do something that’s too physically demanding for other nursing staff, while the female students get to stay.”

Twenty participants opined that inaccurate perceptions of male nursing students in a clinical setting may impact the fair distribution of learning opportunities between genders, with tasks being assigned based on physical strength. Participant 14 recalled the following incident:

“There were three male nursing students in the surgical ward of an acute hospital. I was the slightest in terms of build, and one of the others worked out every day, so he looked the strongest. One day we were observing our mentor—she was an advanced practice nurse—manage high intracranial pressure for a patient with an external ventricular drain, which was a really important lesson. But right in the middle, we were interrupted by a request from a nursing staff to go and help lift an obese patient onto a commode chair. Because my friend was the strongest, he was asked by the mentor to offer help. By the time he got back, the observation had finished, and he’d missed out. Although my friend never spoke about it in clinical areas, he told me that he was pretty angry about the unfairness of it—he was a student just like the rest of us, but he felt he was being treated like a patient-care assistant.”

### 3.3. Male Nursing Students Feel Alienated in the Obstetrics and Gynecology (O&G) Practicum

The theme of alienation emerged most strongly in the obstetrics practicum, which was referred to by the participants as “O&G.” This practicum typically covers rotations in antenatal and postnatal units as well as delivery suites. Respondents (4, 7, 8, and 10) recalled nursing staff commenting negatively about male nursing students’ usefulness from the first day of their arrival. Participant 10 recalled that he was invited by the maternity nurses to look around the ward and read the guidelines. However, he was also told that since he was a male, it was not “convenient” for him to undertake clinical work other than taking patients’ vital signs. For all other patient needs, he was asked to hand over the responsibility to a female nurse. Participant 8 reported that an O&G nurse told him, “you won’t be going into obstetrics in the future because you are a man.” Similarly, Participant 7 reported that several nurses told him that he would “never work in obstetrics” and asked which specialty he would take up when he graduated, assuming that it would not be O&G.

Some participants were also puzzled by the differences between the experiences of male nursing students and male medical students aiming to become gynecologists or obstetricians. While males studying to become physicians seemed to be welcomed in the maternity wards, males studying to become nurses were less so. Participant 5, recalling an interaction he had with midwives, spoke of his confusion as to why they saw the two cohorts differently:

“How do we (as students) differ, fundamentally? I mean, whether we are studying nursing or medicine, all of us are in the wards to get professional training and all of us need supervision from experienced staff so we can learn to provide patient care. Yet, frequently, I (as a male nursing student) was largely excluded from caring for the mums. Male nursing students were often sent out of the patient’s room; only female nursing students could stay with the staff (midwives) and had the opportunity to learn.”

Participants who experienced this sort of treatment were left with feelings of hurt and exclusion, wondering what they had done wrong to be excluded in this way. At least six participants (2, 6, 9, 10, 11, and 12) lamented that while male medical students were invited to observe physical examinations and other clinical procedures, whether carried out by physicians or midwives, male nursing students in a maternity practicum were not.

Four participants (2, 3, 11, and 12) chose the word “alien” to describe their thoughts about the perception of nursing staff from their O&G rotation toward male nursing students. Despite their uniform and name badge, they felt as if their status in the clinical environment was ambiguous, and in some cases, they were treated more like visitors than students. These participants expressed that the midwives who supervised the practicum seemed like operating under restrictive ideas of what was “gender appropriate,” leading to the exclusion of male students from important areas of practice.

### 3.4. More Male Role Models Desired in the Clinical Setting

Participants emphasized that more male role models within clinical settings—particularly as mentors during their practicum—would be an effective way of promoting the position and status of male nurses. Participant 1 noted how useful it had been for him to see that many male nurses had spent many years working within their specialties and achieved senior positions. For him, they served as “living examples” of success in his chosen career. Discussing why more male nurse mentors should be introduced into the practicum, Participant 13 observed that experienced male mentors were not only as effective at supervising clinical practice as female mentors, but they were more likely to understand and address the challenges faced by male nursing students. Discussions with male mentors could offer a useful space to discuss gender-related concerns in clinical practice, and they could also serve as models of socialization within the work environment. Five participants (1, 4, 17, 20, and 22) recommended that more male nurse teachers and nursing students should be recruited, and male role models should be made more visible to ensure that nursing is no longer presented as “women’s work.”

Some participants spoke of their ambition to specialize in high-acuity settings, including intensive care units (ICUs) and emergency departments (EDs). Not only were they confident that they would be able to work effectively in these types of stressful environments, but they also perceived that more males worked in highly technologized areas; therefore, they would be available as mentors, models, and supportive colleagues. Participant 9, for example, believed that male nurses tend to be drawn to critical care because “men are more comfortable with digital machines”; hence, he believed that if he was able to specialize in ICU work, he was more likely to find male role models. Additionally, six participants (4, 5, 6, 8, 10, and 11) were aware of a relatively high number of men in high dependency care unit and ED nursing staff, attributing this to the fact that these areas of practice require quick decision making, which made the areas more suitable for males—as they, in their opinion, tend to immerse themselves in their work and cope better with stress. These participants also noted that they were more likely to find a role model in this type of setting.

## 4. Discussion

Three principal findings emerged from this study. First, there are occasional perceptions of bias against male nursing students within clinical placements (and the bias tends to vary with contextual factors; for example, it may appear more evident in the O&G specialty); second, male students may feel uncomfortable in wards that admit largely female patients (where clinical learning opportunities may not be fairly distributed among students due to gender concerns by nursing staff); and third, they believe that they are sometimes excluded from areas of clinical practice, particularly those relating to women’s health. Thus, the researchers suggest that policy makers in hospitals and nursing schools promote collaboration between male and female nursing staff and students to ensure that male students have the support they need.

This study shows that there is room for improvement in ensuring that male nursing students have access to, and enjoy the benefits of, an effective and inclusive clinical learning environment, particularly with regard to O&G. Previous studies on the experiences of male nursing students undertaking practicum in female wards also showed similar results of perceived alienation and exclusion [18]. The participants in the present study found the exclusion they experienced to be stressful. This finding echoes other studies, which reported that male nursing students often felt excluded from patient care during their training and perceived a certain degree of hostility from female nurses and/or female patients [14,19,20]. A study by Eswi and El Sayed (2011) revealed that more than half of a sample of 60 male nursing students had been excluded from certain aspects of maternal care (53.3%) and believed that their clinical supervisors failed to provide support for male nurses in the ward (50.2%). This experience caused anxiety in 56.6% of respondents, while 38.4% reported having attempted to leave the clinical rotation in maternity care. Hence, we recommend that teaching staff liaise with nursing supervisors to assess the lived experiences of male students and identify where improvements can be made in the treatment of male students entrusted to them for training. Male nursing students may be more likely to receive better treatment in the wards if clinical instructors (or mentors) and nurse supervisors refer to them simply as “nurses” or “nursing students,” without calling patients’ attention to the fact that they are “male nurses” or “male nursing students” [18]. If a female patient requests that only female staff attend to her, this request must be respected; however, it has been found that if attention is not explicitly called to the fact that a male is in the room, it is less likely that such a request will be made [18]. In this way, incorporating and normalizing the presence of male students will enable them to benefit from more learning opportunities. Alternatively, consideration could be given to providing more simulation learning rather than the maternity practicum, as three-quarters of the students surveyed by Eswi and El Sayed (2011) indicated a preference for laboratory or simulation learning over the clinical practice in this area.

Our findings also align with recent studies regarding two other perceptions held by male nursing students, which require the intervention of educators and clinical mentors. First, many have had their right to an in-depth learning experience to a certain extent undercut by the perception that they are useful physical resources (and can be called in for physically demanding situations), even if this may cause them to miss the clinical instruction that their female counterparts are receiving [15,21]. Second, there appears to be a tendency among some female nurses to encourage male students to limit themselves to the care of male patients in mixed wards. Male nursing students interviewed in two recent studies recalled that they were frequently asked to care for male patients, and even when female patients were under their care, the female staff took over duties such as catheter care and toileting [16,19]. Therefore, teaching and mentoring practices should ensure fairness and effectiveness of learning; although the students in the current study did not object to lending a hand when physical strength was required, they did note that learning opportunities should not be compromised.

Furthermore, we recommend that nursing faculty introduce means to ensure that the opportunity to learn from clinical experience is not dependent on gender. Chan et al. (2013) and Juliff et al. (2016) echo these sentiments, indicating a belief among some working nurses that some patients are likely to object to being cared for by a male nursing student. This belief may lead to the situation of male students being exclusively assigned to care for male patients, thus depriving them of important learning opportunities. Nursing faculty should play a key and active role in ensuring that future nursing staff are fully prepared to take care of all patients (both male and female) during their professional practice.

Many participants in this study also referenced the expectation that male nurses are more likely to work in high-acuity areas such as ICUs and EDs, adding to DeVito’s (2016) finding that men are drawn toward advanced practice specialties. This expectation leads to a perception among students that entering these areas will make it easier to find male role models and mentors, and the emphasis on technology and heavy lifting that characterizes these areas of practice is in line with gendered expectations.

Furthermore, the participants agreed that it would be valuable to have more male faculty and role models among clinical supervisors, as this would foster both learning and role socialization. Several studies have reported similar findings [8,22] and recommended having senior male nurses serve as guest speakers during classes, and pairing male students with male nurses in the wards to boost the presence and accessibility of male role models. Alternatively, Hong Kong could follow the example of the American Assembly for Men in Nursing (AAMN)—which has chapters in higher educational institutions across the USA [23]—and set up a male nursing organization to facilitate mentorship opportunities and peer support.

Further, this study (as it relates to the issue of gender diversity) may shed light on the possible need for enhanced health surveillance regarding the mental aspects of health care workers in a working environment. In Hong Kong, although Occupational Safety and Health Ordinance (Cap 509; https://www.hklii.org/eng/hk/legis/ord/509/; accessed at 1 May 2021) exists to ensure the safety and health of all persons when they are at work, there is no monitoring by occupational doctors on the mental health of health care workers (including nurses). Nonetheless, Equal opportunities legislation in Hong Kong includes Sex Discrimination Ordinance (Cap 480) to promote equality of opportunity between men and women. In the sense that nursing students (regardless of gender) are learners under supervision and guidance in their clinical practicum, an inclusive learning environment may also reflect the importance of equal opportunities.

### Limitations and Recommendations

This study used a convenience sample of nursing students from a single baccalaureate prelicensure program in Hong Kong. Responses from a more ethnically, racially, or geographically diverse group may provide different insights to the nursing students’ experiences. Quantitative research methods may be considered to examine the effects of barriers on the outcomes of clinical learning for male prelicensure nursing students. Thus, future research may seek large, diverse samples from multiple geographical areas to quantitatively examine male nursing students’ experiences. More research is recommended on male nursing student experiences to ensure that interventions aimed at improving the clinical learning process and experiences of male nursing students can be identified and tested.

## 5. Conclusions

The findings of this qualitative descriptive study indicate that, occasionally, gender bias exists in the clinical practicum setting of prelicensure undergraduate nursing education, and that there is a need to improve teaching and mentoring practices in clinical settings. Dissatisfaction with clinical training may result when male students perceive that learning opportunities are not fairly distributed among students due to gender concerns by nursing staff. Hence, clinical nurses and mentors should be cognizant of the negative implications associated with the uneven distribution of assignments or work based on gender-related characteristics. Alienation and feelings of exclusion were described by the participants as most strongly in the obstetrics practicum. Importantly, this study added to the literature that the language used by female nurses in their communication (which may not necessarily carry educational value) may aggravate the perceptions of male nursing students toward the inclusiveness of the learning environment. Judgmental claims by staff nurses tend to lead to negative feelings among students, particularly when such claims may devalue and/or trivialize the roles of male nurses in the profession. We thus recommend future interventions to increase staff self-awareness of the use of biased language in communicating with students.

In addition, participants expressed that there is a need for more male role models for role socialization and psychological and learning support for male nursing students. Based on our study findings, we therefore suggest that interventions (such as increasing the accessibility and number of male role models as clinical mentors) may be considered to create a more welcoming and inclusive learning environment for male nursing students at clinical sites. Nevertheless, in the academic/didactic setting, participants acknowledged the considerate attitude of faculty toward gender diversity and found gender bias issues arising from teaching methods to be minimal compared to what they experienced in clinical environments. Finally, in order to promote the learning of male nursing students, faculty and practicing nurses must work together to address potential gender biases in the clinical arena.

## Figures and Tables

**Table 1:** Semi-structured interview guide.

1.	Please tell me your general experience in your clinical placement.
2.	How may different learning opportunities are distributed by nursing staff (who may or may not be your mentor) if both male and female students exist in the same clinical venue and in the same shift?
3.	As a male nursing student, how do you perceive the issues of gender in relation to your placement experiences?
4.	In what way do you think the clinical learning process is influenced by the issues of gender you mentioned previously?
5.	What do you think about the issues of gender in clinical learning as compared to your learning within the School (for example, in classrooms and nursing laboratories)?
6.	Could you suggest some examples to illustrate your placement experiences about which you think the concerned process and/or outcomes may be related to the issues of gender?
7.	Concerning the issues of gender you mentioned previously, what will you recommend to improve the clinical learning experiences of male nursing students?

## Data Availability

The interview guide is provided in a table in the manuscript. To protect participants’ privacy, the transcripts containing private and confidential data, such as the wards and the sites of practice of the participants, will not be made publicly available.
